# Mg(OH)_2_ nanosheets on Ti with immunomodulatory function for orthopedic applications

**DOI:** 10.1093/rb/rbac027

**Published:** 2022-04-29

**Authors:** Yue He, Mengyu Yao, Jielong Zhou, Juning Xie, Changxiang Liang, Dong Yin, Shuaihao Huang, Yu Zhang, Feng Peng, Shi Cheng

**Affiliations:** 1 School of Medicine, South China University of Technology, Guangzhou 510006, China; 2 Medical Research Center, Department of Orthopedics, Guangdong Provincial People’s Hospital, Guangdong Academy of Medical Sciences, Guangzhou 510080, China

**Keywords:** immunomodulation, titanium, nano-magnesium hydroxide, osteogenesis

## Abstract

Macrophages play a vital role for guiding the fate of osteogenesis- related cells. It is well known that nano-topography and bioactive ions can directly enhance osteogenic behavior. However, the effects of nano-structure combined with bioactive ions release on macrophage polarization and the following osteogenesis and angiogenesis are rarely reported. Herein, Mg(OH)_2_ films with nano-sheet structures were constructed on the surface of Ti using hydrothermal treatment. The film presented nano-sheet topography and sustained release of Mg ions. The results of *in vitro* culture of bone marrow-derived macrophages (BMDMs), including PCR, western blot and flow cytometry suggested that the nano-Mg(OH)_2_ films were more favorable for macrophages polarizing to tissue healing M2 phenotype. Moreover, air-pouch model confirmed that the nano-Mg(OH)_2_ film coated Ti would induce milder inflammation and thinner fibrous layer *in vivo*, compared with untreated Ti. Furthermore, macrophages-conditioned culture mediums were collected from nano-Mg(OH)_2_ coated Ti group was superior for the osteogenic behaviors of mice bone marrow stem cells and the angiogenic behaviors of human umbilical vein endothelial cells. With harmonious early inflammatory response and subsequently improved osteogenesis and angiogenesis, the nano-Mg(OH)_2_ coated Ti is promising for orthopedic applications.

## Graphical Abstract

**Figure:**
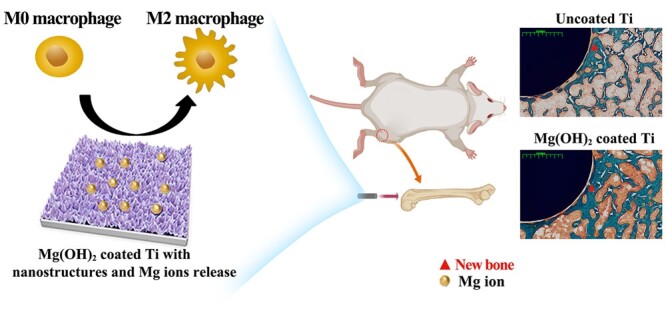

## Introduction

Titanium (Ti)-based implants are one of the most successful orthopedic implants in the past decades [1–3]. Nevertheless, aseptic loosening is a major cause of the failure of implantation, so researchers continuously pursuit for better osteointegration and osteogenesis capabilities of Ti-based bone implants [4–6]. For example, introducing bioactive ions (such as Si, Ca, Mg and Sr) and biomolecules (such as BMP-2, VEGF collagen) onto Ti surface, as well as constructing micro-nano surface are the normal methods to enhance osteogenesis and angiogenesis for improved bone rebuilding [7–12]. Osteoblast, bone stem cells and endothelial cells are involved in the two processes. However, the microenvironment surrounding bone repair area is complicated. The designed biomaterials in laboratory poorly translated to clinical trials, which might be because less attention has been focused on the body defense mechanism. Therefore, more and more studies tried to reveal the cross-talk between the skeletal system and immune system [13–16].

Immune system is the last defense line for humans to resist foreign objects [17]. When a biomaterial is implanted, it will immediately activate immune response [18]. Though various types of immune cells take part in immune response, macrophages plays a critical role in the immune defense and tissue remodeling *via* secreting cytokines. Macrophages are high plastic cells, which can polarize to pro-inflammatory M1 phenotype or anti-inflammatory M2 phenotype [19–21]. Chen *et al*. proposed the concept of ‘osteoimmunomodulation’ in 2016 [14], and pointed out that once macrophages polarize to M1 phenotype excessively, they will secrete a large amount of pro-inflammatory factors, resulting in bone resorption and the formation of fibrous capsule surrounding the implant. On the contrary, when macrophages are activated into M2 phenotype, they will secrete growth factors and cytokines to enhance cell proliferation and differentiation [21]. Typically, macrophages will mainly polarize to M1 phenotype at the initial 3–4 days after implantation, and then change to M2 phenotype for tissue healing. Unfortunately, many bone implants present a prolonged time period of M1 phenotype activation and a delay activation of M2 phenotype, thus resulting in an insufficient early osteointegration. Therefore, orthopedic implants with immunoregulation are potential strategy for clinic applications.

Researchers increasingly tried to regulate immune response of the implants to achieve improved osteogenesis and osteointegration. Lee *et al*. [22] reported that Ca and Sr ions can regulate macrophages’ adhesion and switch its phenotype from M1 to M2. Liu et al. [23] found that a Zn-modified sulfonated polyetheretherketone (PEEK) can induce macrophages to polarize to M2 phenotype and then secreted a set of osteogenic cytokines. Wang *et al*. [24] fabricated a TiO_2_ nanotube on Ti surface to induce macrophage polarization to M2 phenotype. Gao *et al*. [25] constructed a nano-porous surface on PEEK, and found that the surface can suppress the acute inflammatory response of macrophages. Furthermore, using whole-genome expression analysis, they revealed that the inhibition of nucleotide oligomerization domain (NOD)-like receptor and Toll-like receptor signaling pathways were involved in regulating immune function. All these literatures indicated that surface properties, such as ion release and nano-topography, are the important parameters to tune immunomodulatory functions of implants. However, the immune response of a surface combined with nano-structure and bioactive ion release is rarely reported.

Herein, Mg(OH)_2_ films with nano-sheet structures were prepared on the surface of Ti. The adhesion and polarization behaviors of mice bone marrow-derived macrophages (BMDMs) cultured on nano-Mg(OH)_2_ films were studied. After that, immune response of the modified Ti was also studied by mouse air-pouch model. Furthermore, macrophage-mediated osteogenesis of mouse bone marrow stem cell (BMSCs) and angiogenesis of human umbilical vein endothelial cells (HUVECs) were evaluated. The *in vivo* bone regeneration capability of nano-Mg(OH)_2_ coated Ti was also investigated using bone implantation model.

## Experimental section

### Sample preparation and characterization

Ti plates were cut into 10 mm × 10 mm × 1 mm and then ultrasonically cleaned in HF and HNO_3_ mixed aqueous solution as described prior [26]. The plates experienced a hydrothermal treatment in a mixed solution of MgCl_2_ (1.2 mM) and urea (21.6 mM). The reaction was kept at 120°C for 12 h. The samples were labeled as HT-1#. Similarly, the plates treated in solution containing 6 mM of MgCl_2_ and 108 of mM urea were labeled as HT-2#.

The surface morphologies of Ti, HT-1# and HT-2# were observed using a field-emission scanning electron microscope (Fe-SEM; Merlin; Carl Zeiss AG, Germany). A contact angle measuring system (OCA15; Dataphysics, Germany) was used to detect the wettability of the samples.

Ti, HT-1# and HT-2# samples were immersed in deionized water (10 ml/specimen) for various times at 37°C. Mg ions concentration in the extract was quantified via inductively coupled plasma atomic emission spectroscopy (ICM-MS; iCAP RQ; Thermo Fisher Scientific, USA).

### Isolation of mice BMDMs

All animal experimental protocols in this study were conducted following guidance by Animal Ethics Committees of Guangdong provincial people’s hospital (KY-D-2021-365-01). BMDMs were isolated from femurs and tibias of C57BL/6 mouse (male, 3 weeks old). C57BL/6 mouses were sacrificed by cervical dislocation and immersed in 75% ethyl alcohol for 1 h to disinfection. Their femur and tibia bones were separated under aseptic condition and the muscle tissues adhered to the bone were removed with scissors. After cutting off both ends of femur and tibia, a 29-gauge syringe was used to flush out the bone marrow into a 10-cm culture dish containing Roswell Park Memorial Institute (RPMI; Procell, Wuhan, China) medium. The bone marrow was dispersed into cell suspension and centrifuged to harvest cells. The cell pellets were resuspended in 2 ml red blood cell lysing buffer (HyClone, South Logan, UT, USA) and the solution was gently blown several times. After lysing for 5 min, the transparent red solution was centrifuged to collect cell pellets. The supernatant was discarded and the cells were resuspended with RPMI medium containing 10% heat inactivated fetal bovine serum (FBS; Gibco, USA) and 1% antibiotic–antimycotic solution (Gibco, USA) and cultured in a 10 cm dish. After 1 day, the suspended cells were collected and cultured in a new dish. M-CSF (25 ng/ml, peprotech, Rocky Hill, NJ, USA) was added into the culture medium to their final concentration to stimulate maturation of BMDMs. After growth into 80% confluence, cells were scraped with cell scrapers for the following experiments.

### Cell viability evaluation of BMDMs

#### Cell proliferation and live/dead staining

The cells were seeded on each sample with a concentration of 1 × 10^4^ cell/specimen. At scheduled time (Days 1, 4 and 7), CCK-8 reagent was added into each well. After culturing for 1.5 h, the OD_450nm_ value of the supernatant was measured using a microplate reader (Thermofisher, USA). For live/dead staining, the cells were cultured for 1 and 3 days. Then the cells were washed with PBS twice. Calcein AM and propidium iodide (Sigma, USA) were dissolved in RPMI according to the manuscript’s instruction. The prepared working solution was used to stain the cells for 2 h avoiding light. After rinsing with PBS, the live and dead cells were observed under a fluorescence microscope (Olympus IX 71, Olympus, Japan).

#### Flow cytometric analysis of apoptosis

BMDMs were seeded on the samples for 3 days. The cells were detached by Trypsin-EDTA and resuspended using 10-fold diluted Annexin V Binding Solution (Dojindo, AD10, Japan) to make a final cell concentration of 1 × 10^6^ cells/ml. Then, the cells were added with Annexin V. After incubating for 10 min, the cell suspension was added with 5 μl of propidium iodide (PI) solution. This solution was detected using FACScan flow cytometer detection (Beckman, USA). The result of cytometric detection was calculated by Flow Jo software.

#### Cell adhesion

BMDMs were seeded on each sample for 12 h. Then, the cells were fixed with 4% paraformaldehyde (PFA) and subsequently treated with triton X-100. Thereafter, the cytoskeletons and nuclei were stained with phalloidin and 4',6-diamidino-2-phenylindole (DAPI), respectively. The cells were visualized using fluorescence microscope (Olympus IX 71, Olympus, Japan) at three random views.

### Polarization evaluation of BMDMs

#### Quantitative Real-Time PCR (RT-PCR) analysis

BMDMs were seeded on each sample for 1 and 3 days. Then, the RNA was extracted and detected using Trizol and Nanodrop 2000, respectively. Complementary DNA (cDNA) was reverse-transcribed with the First Strand cDNA Synthesis Supermix Kit (Yeasen, China). Quantitative Real-time PCR (qRT-PCR) reactions were performed using mixed working solution containing SYBR Green Mastermix, primers and synthesized cDNA. The gene expression was calculated using the 2^−△△^CT method. The used sequences in this study are listed in Supplementary Table S1.

#### Western blotting (WB) analysis

BMDMs were seeded on each sample for 3 days. Then, the cells were lysed using radio immunoprecipitation assay (RIPA) lysis buffer (Beyotime, Shanghai, China). The concentration of harvested total protein in each group was determined through a bicinchoninic acid (BCA) assay kit (Beyotime). Then, 30 μg of total protein of each group was added to corresponding lanes. Target protein was separated using sodium dodecyl sulfate polyacrylamide gel electrophoresis (SDS-PAGE). After electrophoresis, the proteins were transferred onto polyvinylidene difluoride (PVDF) membranes, blocked with 5% BSA in tris buffered saline with tween (TBST), and incubated with primary antibodies including anti-CD206 (1:1000, abcam, ab64693), anti-iNOS (1:200, invitrogen, PA1-036), and anti-β actin (1:2000, abcam, ab8227) at 4°C overnight. Then, they were incubated with horseradish peroxidase (HRP)-conjugated secondary antibodies (1:5000, abcam, ab205718) and observed using enhanced chemiluminescence reagent (Thermo Fisher Scientific, USA).

#### Flow cytometric analysis

BMDMs were seeded on each sample for 3 days. Then, the expressions of CD86 and CD206 were analyzed according to the Section ‘Cell adhesion’.

### Air-pouch model evaluation

Sprague-Dawley (SD) rats (male, 4 weeks old) were injected with sterile air (5 ml) subcutaneously and a second injection (3 ml) 4 days later according to the previous study [23, 27]. After another 2 days, the mice were anesthetized using 1% pentobarbital sodium solution (1 ml/100 g). The fur was shaved thoroughly and the skin was sterilized. One specimen was inserted into each pouch. After implantation for 1 and 4 days, the tissues surrounding the implants were fixed in 4% PFA, embedded in paraffin and cut into sections. Then, the sections were stained with hematoxylin and eosin (H&E) and immunofluorescence (CD206, iNOS) to evaluate the inflammatory reaction of the skin.

### Acquisition of macrophage-conditioned culture medium

1 × 10^4^ BMDMs were seeded on each sample in 6-well plate with 5 ml complete culture medium. The culture medium was refreshed after 1 day for cell adhesion. After culturing for another 2 days, the culture medium was collected and centrifuged. The supernatant was labeled as macrophage-conditioned medium (MCM). For osteogenic differentiation behavior evaluations, MCM was added with 10 mM β-glycerophosphate, 100 nM dexamethasone and 50 mM ascorbate and glutamine. For angiogenesis test, MCM was added with complete endothelial cell culture medium (v/v = 1:1).

### Osteogenic differentiation of BMSCs cultured in MCM

BMSCs were cultured in 24-well for 1 day, and then the cultured mediums were removed and each well was added with MCM.

#### Alkaline phosphatase (ALP) activity

BMSCs were cultured in MCM for 14 days. Then, the cells were fixed in 4% paraformaldehyde, stained with 5-bromo-4-chloro-3-indolyl phosphate P-toluidine salt/nitro blue tetrazolium (BCIP/NBT ALP) color development kit (Beyotime, China), and observed using optical microscope (Olympus, Japan). On the other hand, the cells were lysed in RIPA, and ALP activity was quantified using p-nitrophenyl phosphate (pNPP). The results were normalized to protein calculated using BCA assay kit.

#### Extracellular matrix mineralization (ECM) analysis

After culturing for 14 days in MCM, the cells were fixed in 4% PFA and stained with Alizarin Red. After rinsing with PBS, the cells were captured under an optical microscope. Thereafter, Alizarin Red was dissolved with 10% cetylpyridinium chloride (Sigma, USA) and the absorbance was measured at 620 nm.

#### RT-PCR assay

After culturing for 3 days in MCM, the expressions of osteocalcin (OCN), osteopontin (OPN) and ALP were analyzed using RT-PCR according to the Section ‘Quantitative Real-Time PCR (RT-PCR) analysis’.

#### WB assay

After culturing for 7 days in MCM, the cells were lysed with RIPA to extract total protein for WB assay. Osteogenic proteins alkaline ALP and Runt-related transcription factor 2 (RUNX-2) were detected. Primary antibodies against ALP (ab229126, 1:1000) and RUNX-2 (ab23981, 1:1000) were purchased from Abcam. The procedure of WB was described in Section ‘Western blotting (WB) analysis’.

### Angiogenic behaviors of HUVECs cultured in MCM

#### RT-PCR assay

HUVECs were seeded in 6-well plates. After culturing for 1 day, the culture medium was removed, and added with MCM. Three days later, vascular endothelial growth factor (VEGF), vascular endothelial growth factor receptor 2 (KDR) and hypoxia inducible factor-alpha (HIF-α) genes were analyzed according to the procedure described in Section ‘Quantitative Real-Time PCR (RT-PCR) analysis’.

#### Tube formation assay

Matrigel (Coring, 356234) was thawed out at 4°C and added into 48-well plate at 50 μl/well. Then the Matrigel coated plate was incubated at 37°C for solidification of the Matrigel. Thereafter, 1 × 10^4^ HUVECs were seeded at each well, and cultured with MCM. After incubation for 12 h, tube structures were observed under a microscope and analyzed using image J software.

### In vivo bone regeneration study

Sprague-Dawley rats (male, 5 weeks old) were anesthetized using 3% pentobarbital sodium. A hole was drilled along the long femur axis. After that, the sample (2 mm in diameter, 8 mm in length) was implanted and the wound was gently sutured layer by layer. Benzylpenicillin sodium (400 000 units/kg) was injected near the femur consecutively for 3 days. The rats were sacrificed after 8 weeks of implantation. The femurs were collected and subjected to methamphetamine blue staining.

### Data analysis

Data were expressed as mean ± standard deviations (*n* = 4) and analyzed using GraphPad Instant Software. The outlier data for each experiment was removed before analysis. One-way analysis of variance (ANOVA) was applied. *P *<* *0.05 was considered to be statistically significant.

## Results and discussion

### Surface characterization of nano-Mg(OH)_2_ coated Ti

**Figure 1.**
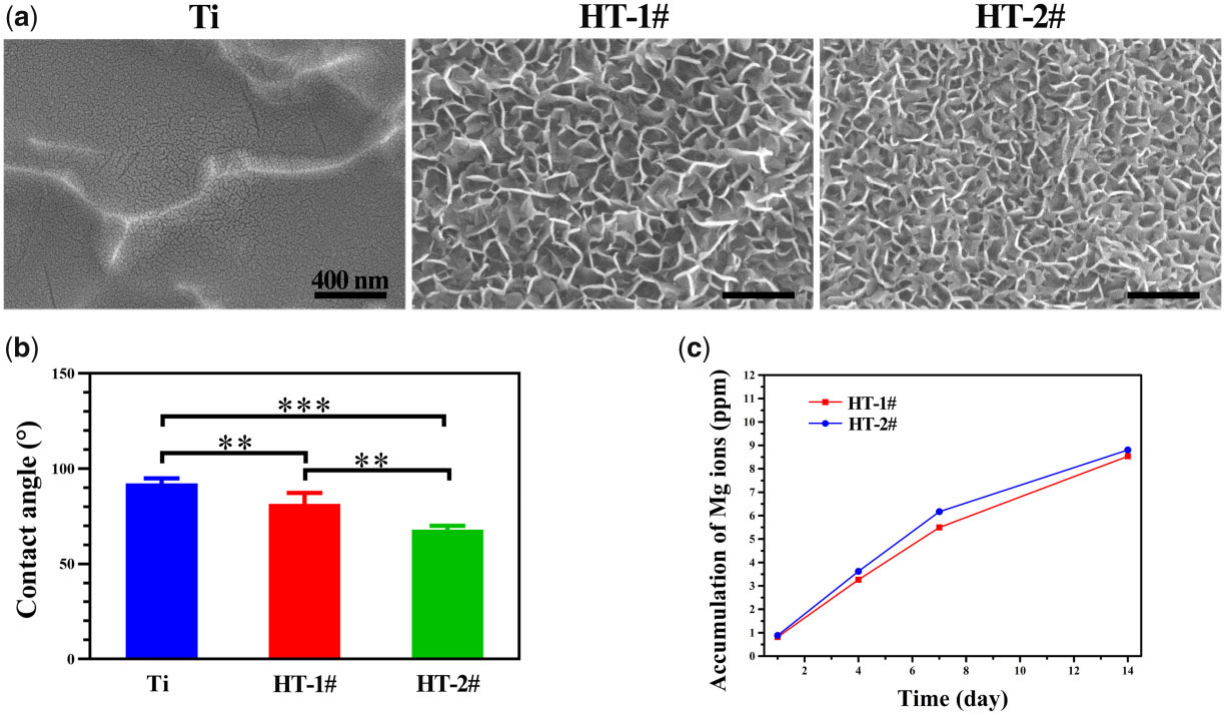
Surface morphology (**a**) and water contact angle (**b**) of Ti, HT-1# and HT-2#. Accumulative release of Mg ions from HT-1# and HT-2# (**c**)

The surface views of Ti, HT-1# and HT-2# are shown in Fig. 1a. The uncoated Ti showed a relative flat surface with nano-cracks. After hydrothermal treatment, both HT-1# and HT-2# were fully and evenly covered by nano-sheet structures. The nanosheets on Ti-1# surface were larger than that on HT-2# surface. The urea in hydrothermal solution would hydrolyze to form NH_3_ and CO_2_, so reactions in the Telfon-line stainless steel contained: CO(NH_2_)_2_ + H_2_O → 2NH_3_ + CO_2_, NH_3_ + H_2_O → ${\text{NH}}_{4}^{+}$ + OH^−^, and Mg^2+^ + 2OH^−^ → Mg(OH)_2_. Therefore, it is believed that the nanosheets, which is the typical morphology of metal hydroxides, on the sample’ surfaces were Mg(OH)_2_ products. The XRD patterns shown in Supplementary Fig. S1 suggested that only characteristic peaks of Ti but no characteristic peaks of Mg(OH)_2_ were detected for the coated samples, which might be because the Mg(OH)_2_ films were too thin to be detected. Figure 1b shows the water contact angles of various samples. The Ti surface showed a contact angle of nearly 90°. Comparing to Ti, HT-1# and HT-2# samples presented decreased contact angles, which can be ascribed to the hydrophilic property of nano-Mg(OH)_2_ on their surfaces. As the Mg(OH)_2_ breaks down in liquid, Mg^2+^ would release from the coated samples. The accumulative releases of Mg ions from HT-1# and HT-2# samples are displayed in Fig. 1c. Both the samples exhibited a slow and sustained release of Mg^2+^, and Mg^2+^ released from HT-2# sample was a few higher than that from HT-1# sample.

### Cell compatibility of nano-Mg(OH)_2_ coated Ti

**Figure 2.**
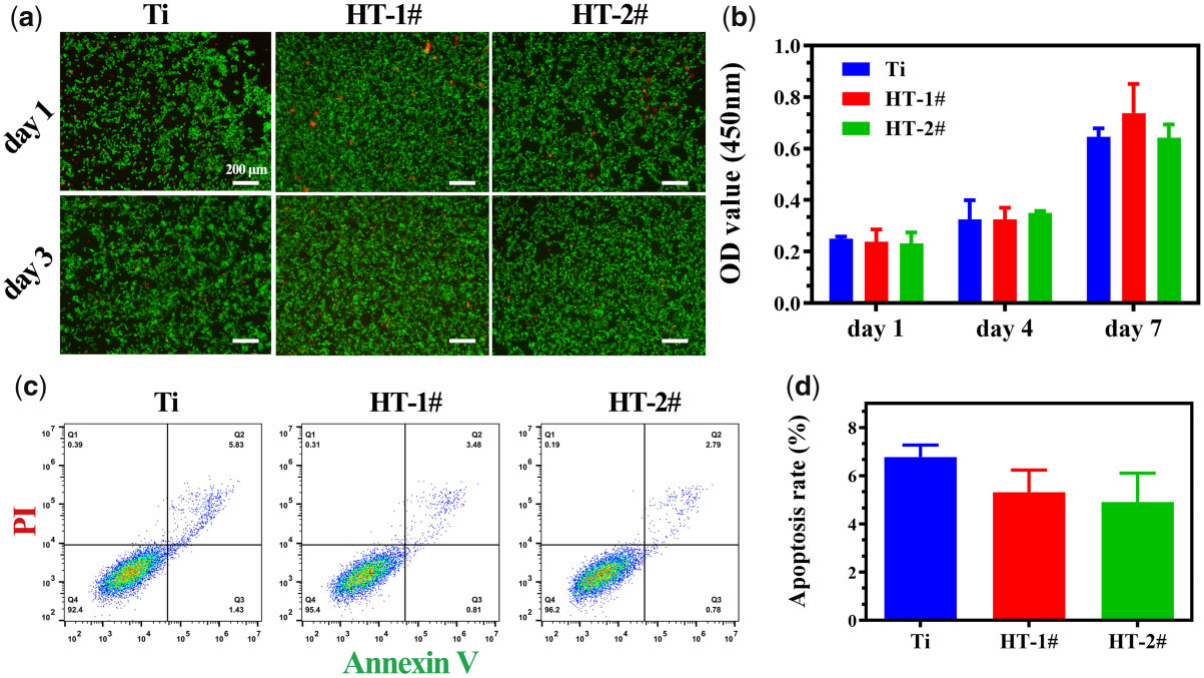
Live/dead staining (**a**) and cell proliferation (**b**) of BMDMs after cultured on Ti, HT-1# and HT-2# for various time. Flow cytometry analyses of apoptosis marker on macrophages (**c**) and corresponding quantitative result (**d**)

**Figure 3.**
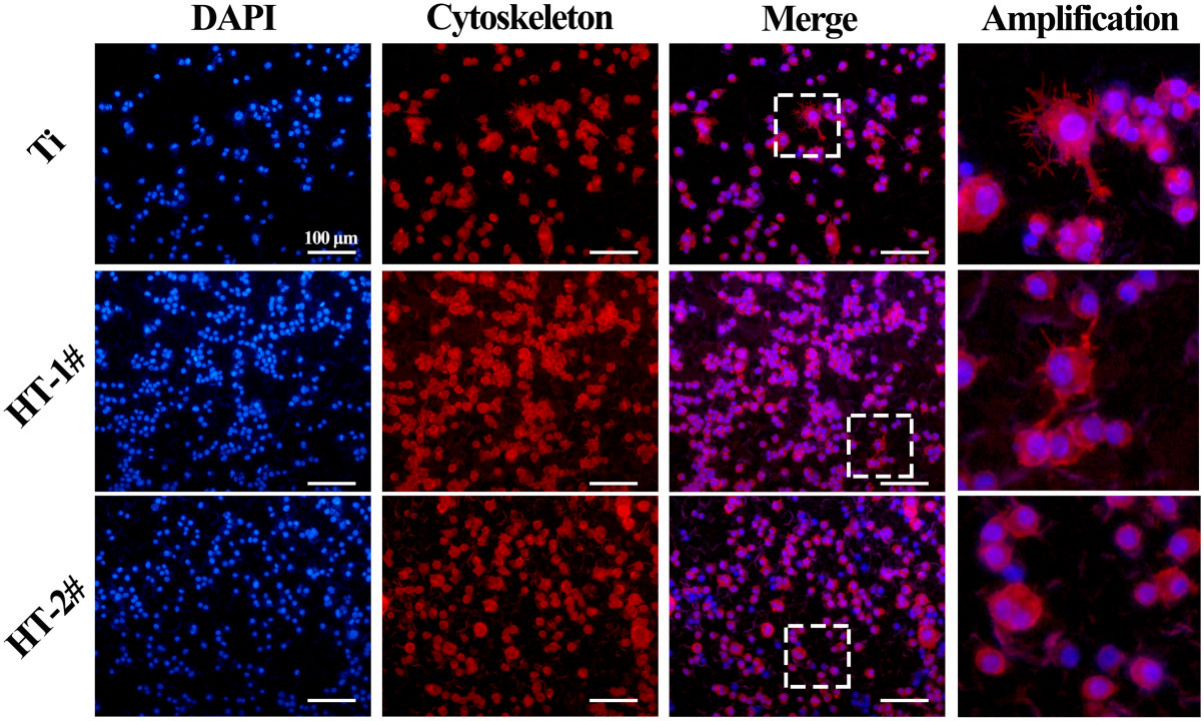
Adhesion of BMDMs after cultured on Ti, HT-1# and HT-2# surfaces for 12 h

Figure 2a presents the live/dead staining results of BMDMs. Only few dead cells were observed on all the three samples both on Days 1 and 3. The results of CCK-8 (Fig. 2b) revealed that BMDMs showed increasing proliferation on the three samples, but no significant differences were detected between the three groups at all the time points. Furthermore, the apoptosis test by flow cytometric analysis (Fig. 2c and d) showed a larger ratio of apoptosis cells on Ti surface than that on HT-1# and HT-2# surfaces, but no statistic difference was observed. These results demonstrated that the prepared nano-Mg(OH)_2_ coated Ti samples showed equally good cytocompatibility with uncoated Ti. We also evaluated the morphology of BMDMs after culturing on various samples for 12 h (Fig. 3 and Supplementary Fig. S2). The cells on Ti showed many filopodia, while the cells on HT-1# showed significantly less filopodia than that on Ti surface. Notably, the cells on HT-2# surface showed round shape. It was reported that a round and no spreading morphology of macrophage tends to polarize more to M2 phenotype [27].

### *In vitro* polarization of mBMDM cultured on nano-Mg(OH)_2_ coated Ti

**Figure 4.**
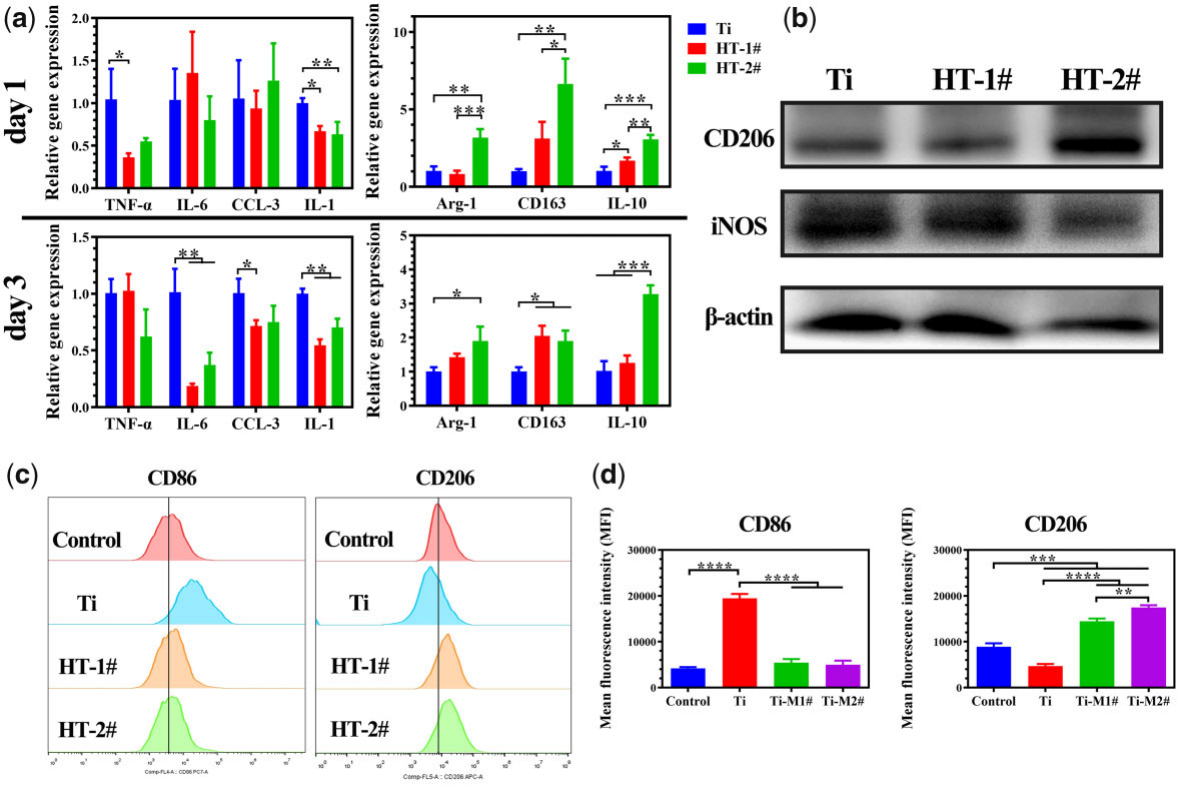
Relative mRNA expression of M1 (TNF-α, IL-6, CCL-3, IL-1) and M2 (arg-1, CD163, IL-10) related genes in BMDM after cultured on various samples (**a**). Expression of CD206 (M2 marker) and iNOS (M1 marker) proteins in BMDMs after cultured on various samples for 3 days (**b**). Mean fluorescence intensity of CD86 (M1 marker) and CD206 (M2 marker) in BMDMs determined by flow cytometry (**c**)

When macrophage polarizes to M1 phenotype, the cells will show high expression in genes, such as TNF-α, IL-6, CCL-3, IL-1, etc.; or polarize to M2 phenotype, the cells will show high expression in genes, such as Arg-1, CD163, IL-10 and so on [28]. The expression levels of immune-related genes in BMDMs were detected (Fig. 4a). On Day 1, no statistic difference was found among the three groups for the expression of IL-6 and CCL-3. But Mg(OH)_2_ coated groups exhibited lower expression of TNF-α and IL-1 genes. The cells cultured on HT-2# sample showed significantly higher expression in Arg-1, CD163 and IL-10 genes. When the incubation time increased to 3 days, IL-6, CCL-3 and IL-1 were all lower expressed in HT-1# and HT-2# groups. Meanwhile, HT-2# group still showed higher expression in the three M2-related genes than Ti group, whereas HT-1# only showed significantly higher expression of CD163 than Ti group. Notably, for both of the two time points, the cells cultured on HT-1# and HT-2# showed few differences in the expression of M1-related genes, but HT-2# showed significantly higher expression of M2-related genes (Arg-1, CD163 and IL-10 on Day 1; IL-10 on Day 3). The above PCR results suggested that HT-2# sample can best induce M2-polarization of macrophages. CD206 protein is highly expressed in M2 and iNOS protein is highly expressed in M1 phenotype. Hence, we further investigated the expression of CD206 and iNOS in BMDMs. As shown in Fig. 4b and Supplementary Fig. S3, the cells cultured on HT-2# sample showed highest expression of CD206 and lowest expression of iNOS. The immunofluorescence staining results of CD206 and iNOS confirmed that CD206 was highest expressed and iNOS was lowest expressed on cell membrane when cultured on HT-2# sample (Supplementary Figs S4 and S5). Furthermore, flow cytometry was used to analyze the expression patterns of membrane proteins. CD206 protein was stained to label M2 polarized BMDMs and CD86 was stained to label M1 polarized BMDMs. As shown in Fig. 4c, the cells cultured on Ti showed significantly higher expression of CD86 and lower expression of CD206 than on the control group. For HT-1# and HT-2# groups, the cells displayed the same level expression of CD86 with the control group, but significantly higher expression of CD206. In all, from proteins to genes, the results of PCR, WB, immunofluorescence staining and flow cytometry demonstrated that BMDMs cultured on nano-Mg(OH)_2_ modified surface tends to polarize more to M2 phenotype, which is conducive to release cytokines for tissue repair.

Immune response predominates at the early stage of implantation. A fast and quick M1 polarization of macrophage and then followed by a tissue-repair M2 polarization of macrophage is known to be good for bone regeneration. The surface property of implanted biomaterial can directly guide cell fate. Therefore, many researchers tried to modulate surface characterization (such as topography and release of bioactive ions) to achieve proper immune response. Both Qiao *et al*. and Li *et al*. [29, 30] fabricated Mg-dope TiO_2_ surface and found that the introduction of Mg can downregulate the expression of pro-inflammation-related genes, while upregulating the anti-inflammation-related genes. Mg ions were also incorporated into poly(lactic-co-glycolic acid)/calcium phosphate tribasic (PLGA/TCP) porous scaffold and graphene oxide nanoscrolls decorated biomimetic scaffolds to guide macrophage for better bone regeneration [31, 32]. Moreover, there is evidence revealing that Mg ions below 100 mg/l present osteoimmunomodulatory function for bone regeneration [33]. Except bioactive metal ions, surface topography also can influence polarization fate of macrophage. Chen *et al*. [27] found that nano-structure with a lower aspect ratio possess a higher elastic modulus, thus not conducive to macrophage adhesion and spreading, leading to a moderate immune response. Ni *et al*. [34] found that nano-sized concave pit/convex dot microarray can guide immunomodulatory osteogenesis and Yang *et al*. [35] reported that micro/nano hierarchical hydroxyapatite can stimulate osteogenesis via macrophage modulation. In this study, to utilize the immunomodulatory function of Mg ions and topography, we used Mg to fabricate nano-structures on Ti, namely nano-Mg(OH)_2_ film. The nano-Mg(OH)_2_ modified Ti not only possessed a rough topography (Fig. 1a), but also showed a slow and sustained release of Mg ions (Fig. 1c). Because Mg(OH)_2_ is a biocompatibility substance, the nano-Mg(OH)_2_ coated Ti exhibited good cytocompatibility (Fig. 2). Furthermore, stimulated by the nano-sheet topography and Mg ions, BMDMs cultured on nano-Mg(OH)_2_ coated Ti showed enhanced M2 polarization and suppressed M1 polarization (Fig. 4), indicating that nano-Mg(OH)_2_ films possessed proper immune regulation function for bone tissue reconstruction.

### *In vivo* immune response of nano-Mg(OH)_2_ coated Ti

**Figure 5.**
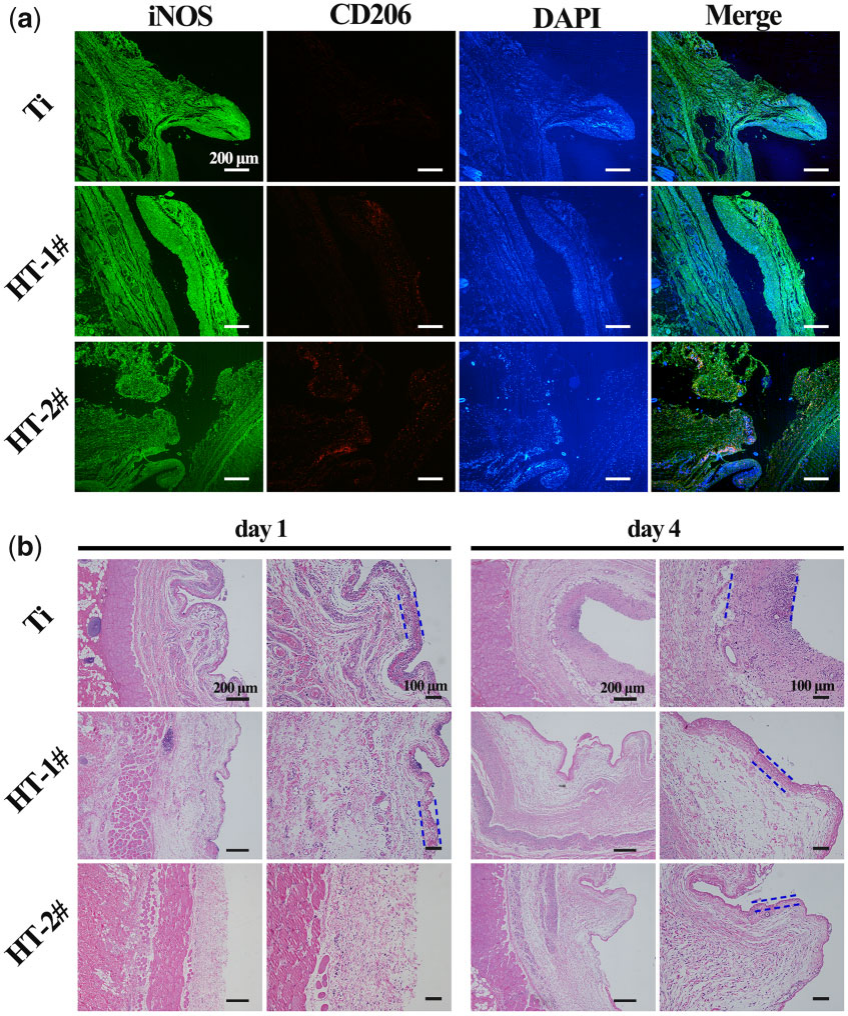
Immunofluorescence images of iNOS and CD206 in tissues adjacent to various samples (**a**). H&E staining of the tissues adjacent to various samples (**b**). The blue dotted lines indicate the fibrous layers

Considering that the actually immune environment in body was totally different with the *in vitro* two-dimensional culture, an air-pouch model was established to investigate *in vivo* immune response of nano-Mg(OH)_2_ modified Ti. Figure 5a shows immunofluorescence staining of the tissues surrounding the implants after being implanted for 4 days, and the high-magnification images are shown in Supplementary Fig. S6. Ti and HT-1# groups exhibited brighter stained color of iNOS (green), whereas HT-2# group showed highest expression of CD026 (red). The tissues were also subjected to H&E staining. As shown in Fig. 5b, on Day 1, obvious fibrous layers were observed for Ti and HT-1# groups. There were few inflammatory cells observed for HT-2# groups. On Day 4, the fibrous layers of Ti and HT-1# groups grew thicker and there was fibrous layers observed for HT-2# group. Nevertheless, the fibrous layer for HT-2# group was thinnest among the three groups. Both the results of immunofluorescence and H&E staining confirmed that HT-2# sample induce milder inflammation, which is in line with the *in vitro* results.

### Osteogenesis and angiogenesis inducing ability of MCM

**Figure 6.**
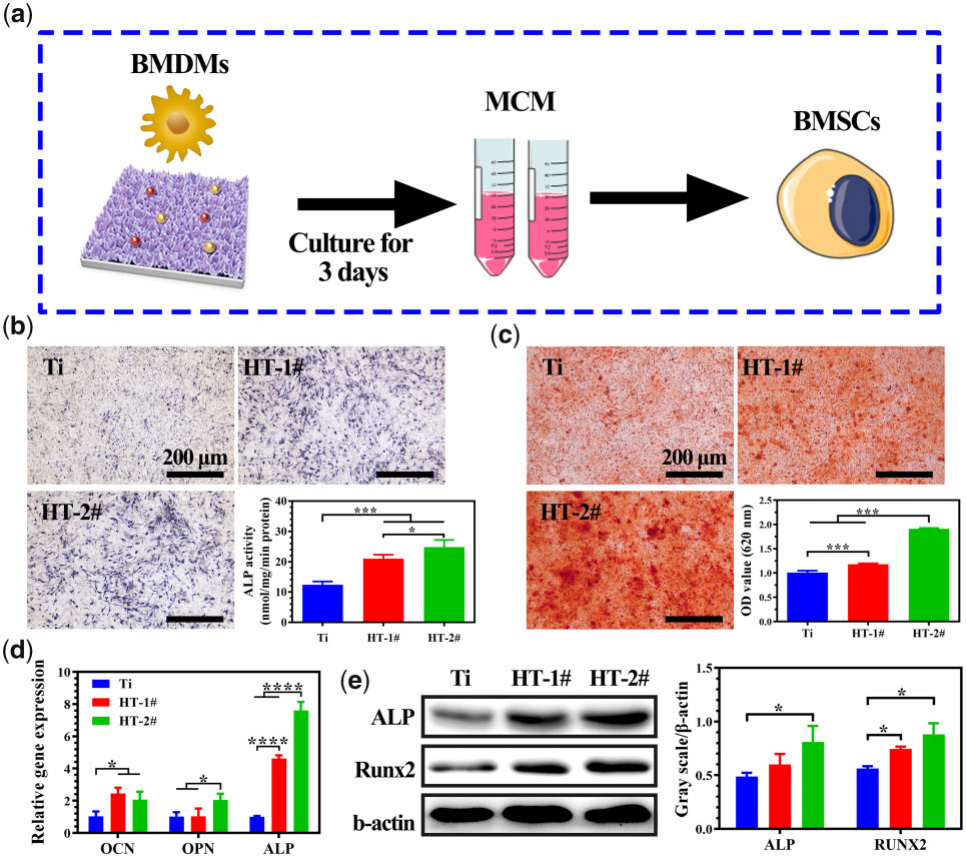
Scheme of experiment process (**a**). ALP activity (**b**) and ECM mineralization (**c**) analysis of BMSCs cultured. Relative mRNA expression of osteogenesis-related genes in BMSCs (**d**). Expression of ALP and RUNX2 proteins in BMSCs and corresponding quantitative analysis (**e**)

**Figure 7.**
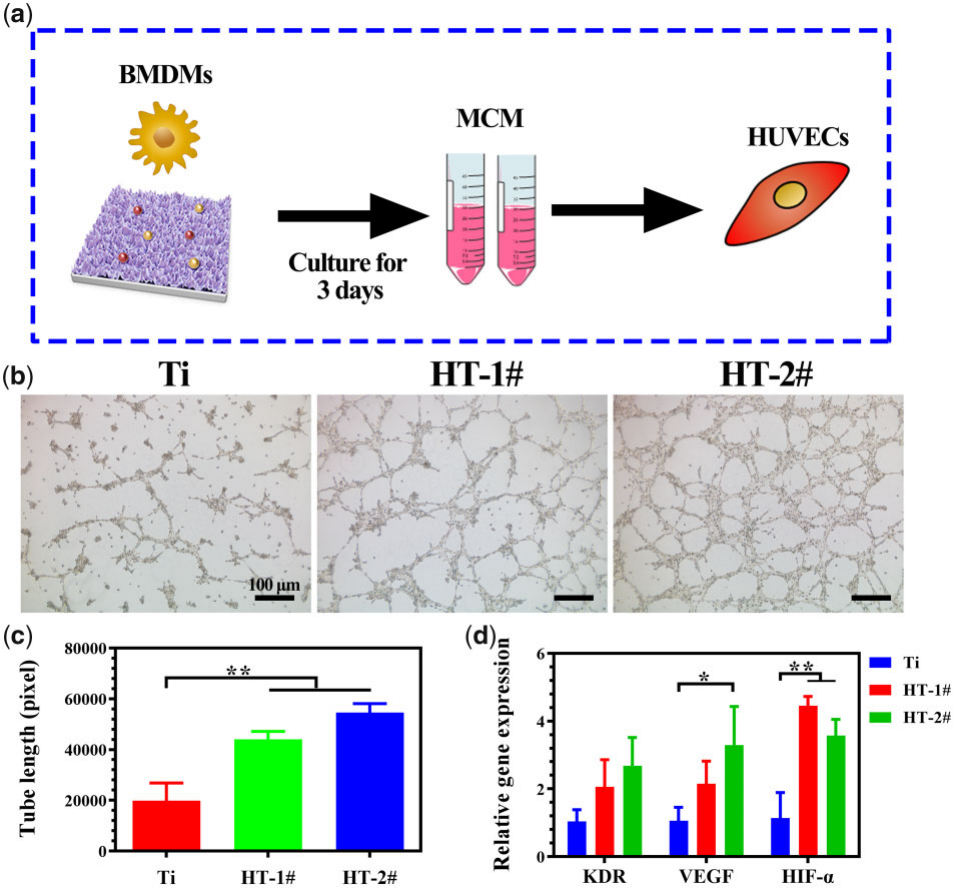
Scheme of experiment process (**a**). Tube formation ability of HUVECs after cultured in MCM for 12 h (**b**). The calculated tube lengths of the formed vessel (**c**). Relative mRNA expression of angiogenesis-related genes in HUVECs after cultured in MCM for 3 days (**d**)

For bone remodeling, the sufficient formation of blood on the injured area can guarantee the delivery of growth factor and nutrients for new bone growth [36]. Hence, both the osteogenic and angiogenic behaviors after immune reaction are vital for the fast and better bone reconstruction. In this study, we investigated the osteogenesis and angiogenesis behaviors by culturing related cells in MCM to mimic this process *in vitro* (Figs 6a and 7a) [37, 38].

The results of ALP activity analysis are shown in Fig. 6b. More bonded dyes were observed for the BMSCs cultured in MCM of HT-1# and HT-2# than that in Ti MCM. Quantitative analysis suggested that HT-2# group exhibited significantly higher ALP activity than the other two groups. Figure 6c shows the staining result of mineralized nodules and corresponding quantitative analysis. It can be seen that HT-2# groups demonstrated significantly more mineralized nodules than the other two groups. The osteogenic differentiation of BMSCs was also evaluated in gene level. As shown in Fig. 6d, compared with Ti group, HT-1# group showed significantly higher expression of OCN and ALP genes, while all the three genes (OCN, OPN and ALP) were upregulated in HT-2# group. Furthermore, the ALP and RUNX2 proteins were also highest expressed in HT-2# group (Fig. 6e). All the above results demonstrated the best osteogenic differentiation of BMSCs cultured in MCM extracted from HT-2# group.

Figure 7b shows the typical images of tube formation test. Few connected tubes were observed for Ti group. However, mature tune connections were observed in HT-1# and HT-2# groups. The quantitative tube length results confirmed that the MCM obtained by HT-1# and HT-2# groups was better for the angiogenic behavior of HUVECs (Fig. 7c). Figure 7d shows the angiogenesis-related genes expressed by HUVECs. The cells cultured in MCM of HT-1# and HT-2# groups showed higher expression of KDR, but no statistic difference was detected. For the genes of VEGF and HIF-α, HT-2# group was significantly higher expressed than Ti group. Both the results of PCR and tube formation confirmed the best angiogenesis ability of HUVECs cultured in MCM extracted from HT-2# group.

It is well recognized that macrophage activated into M1 phenotype will secrete pro-inflammatory cytokines to induce osteoclastogenesis, leading to bone resorption [14]. On the contrary, when activated into M2 phenotype, it tends to secrete osteogenic cytokines (such as BMP2 and VEGF) to enhance osteogenesis process. Our cell and animal data revealed that Mg(OH)_2_ modified Ti can better induce anti-inflammatory M2 polarization, thus the MCM extracted from HT-1# and HT-2# contained more osteogenic and angiogenic cytokines, which finally induce better osteogenic differentiation of BMSCs and angiogenic differentiation of HUVECs.

**Figure 8.**
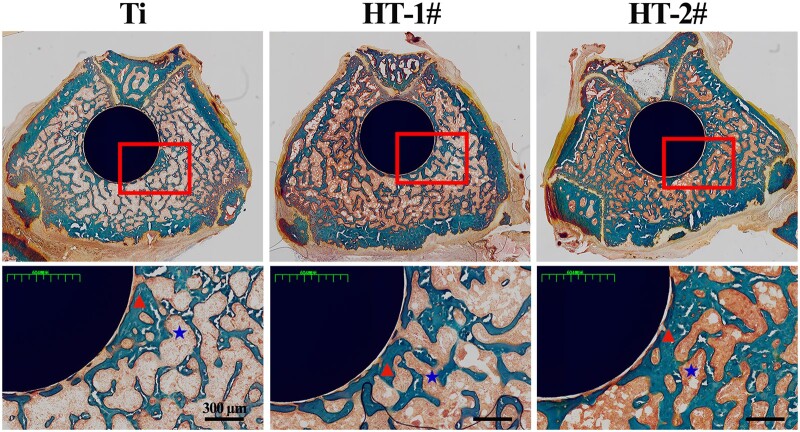
Methamphetamine blue staining of the obtained femur bone after implantation for 8 weeks

The osteointegration capability of Mg(OH)_2_ modified Ti was *in vivo* investigated via implanting the samples into rat femur for 8 weeks as shown in Fig. 8. Compared with Mg(OH)_2_ coated Ti, more cavities (indicated by blue asterisk) were observed for Ti group. In addition, a large area of newly formed bone (indicated by red triangle) was detected surrounding HT-2# implant, and the bone was also closely adhered on the implants surface, suggesting that HT-2# induced better osteointegration.

## Conclusions

In this study, Mg(OH)_2_ films with nano-structures and ability to sustained release of Mg ion were constructed on Ti via hydrothermal treatment. *In vitro* cell culture suggested that nano-Mg(OH)_2_ film can enhance the M2 polarization of mBMDM and suppress its M1 polarization. Air-pouch model experiment confirmed that nano-Mg(OH)_2_ modified Ti can reduce inflammatory cell infiltration and inhibit fibrous capsule formation. Furthermore, the macrophage-conditioned culture medium was favorable for the osteogenic differentiation of mBMSCs and the angiogenic differentiation of HUVECs. Finally, the *in vivo* results of bone implantation suggested that nano-Mg(OH)_2_ modified Ti showed improved osteointegration. With proper immunomodulatory property and following enhanced bone remodeling, the nano-Mg(OH)_2_ modified Ti is promising for orthopedic applications.

## Supplementary data

Supplementary data are available at *REGBIO* online.

## Funding

This work was financially supported by the National Natural Science Foundation of China (52001076), the Scientific and Technological Projects of Guangzhou, China (202102020431), the NSFC Incubation Project of Guangdong Provincial People’s Hospital (KY0120220044) and the Guangdong Basic and Applied Basic Research Foundation (2021A1515110135).

*Conflict of interest statement*. The authors declare no conflict of interest.

## Supplementary Material

rbac027_Supplementary_Data
