# Supplementary material for: Mg(OH)2 nanosheets on Ti with immunomodulatory function for orthopedic applications
Source: Regen Biomater. 2022 Apr 29;9:rbac027. doi: 10.1093/rb/rbac027 (PMC9113411; doi:10.1093/rb/rbac027)
Supplement: rbac027_Supplementary_Data [file rbac027_supplementary_data.docx]

**Mg(OH)_2_ nanosheets on Ti with immunomodulatory function for orthopedic applications**

Yue He^a,b,1^, Mengyu Yao^b,1^, Jielong Zhou^b,1^, Juning Xie^a,b^, Changxiang Liang^b^, Dong Yin^b^, Shuaihao Huang^b^, Yu Zhang^b,a,*^, Feng Peng^b,*^, Shi Cheng^b,*^

*^a^* *School of medicine, South china university of technology, Guangzhou 510006, China*

*^b^ Medical Research Center, Department of Orthopedics, Guangdong Provincial People's Hospital, Guangdong Academy of Medical Sciences, Guangzhou, 510080, China*

***Corresponding Author:**

E-mail addresses: zhangyu@gdph.org.cn (Y Zhang), pengfeng@gdph.org.cn (F Peng), [chengshi@gdph.org.cn](mailto:chengshi@gdph.org.cn) (S Cheng)

**^1^These authors contributed equally to this work.**

**Table S1. The primer sequence for target genes used in this study.**

| genes | Forward sequence | Reverse sequence |
| --- | --- | --- |
| IL-1 | CCCAACTGGTACATCAGCACCTC | GACACGGATTCCATGGTGAAGTC |
| TNF-α | GGACTAGCCAGGAGGGAGAA | CGCGGATCATGCTTTCTGTG |
| CCL-3 | TGTACCATGACACTCTGCAAC | CAACGATGAATTGGCGTGGAA |
| IL-10 | GCTCTTACTGACTGGCATGAG | CGCAGCTCTAGGAGCATGTG |
| CD163 | ATGGGTGGACACAGAATGGTT | CAGGAGCGTTAGTGACAGCAG |
| IL-6 | CTGCAAGAGACTTCCATCCAG | AGTGGTATAGACAGGTCTGTTGG |
| Arg-1 | CTCCAAGCCAAAGTCCTTAGAG | GGAGCTGTCATTAGGGACATCA |
| KDR | AGCAGGATGGCAAAGACTAC | TACTTCCTCCTCCTCCATACAG |
| VEGF | CAGGACATTGCTGTGCTTTG | CTCAGAAGCAGGTGAGAGTAAG |
| HIF-α | TCTACCAGTTGCAGCCTGAC | GTTCCCTTCCTCCTTGATTT |
| OCN | AGACTCCGGCGCTACCTT | CTCGTCACAAGCAGGGTTAAG |
| OPN | TTCTGAGGGACTAACTACGACC | GGCTGTAAAGCTTCTTCTCCTCTG |
| ALP | ACTCAGGGCAATGAGGTCAC | CACCCGAGTGGTAGTCACAA |
| GAPDH (mouse) | TTCCAGGAGCGAGACCCCACTA | GGGCGGAGATGATGACCCTTTT |
| GAPDH (human) | CAAGAGCACAAGAGGAAGAGAG | CTACATGGCAACTGTGAGGAG |





**Figure S1.** XRD patterns for various samples


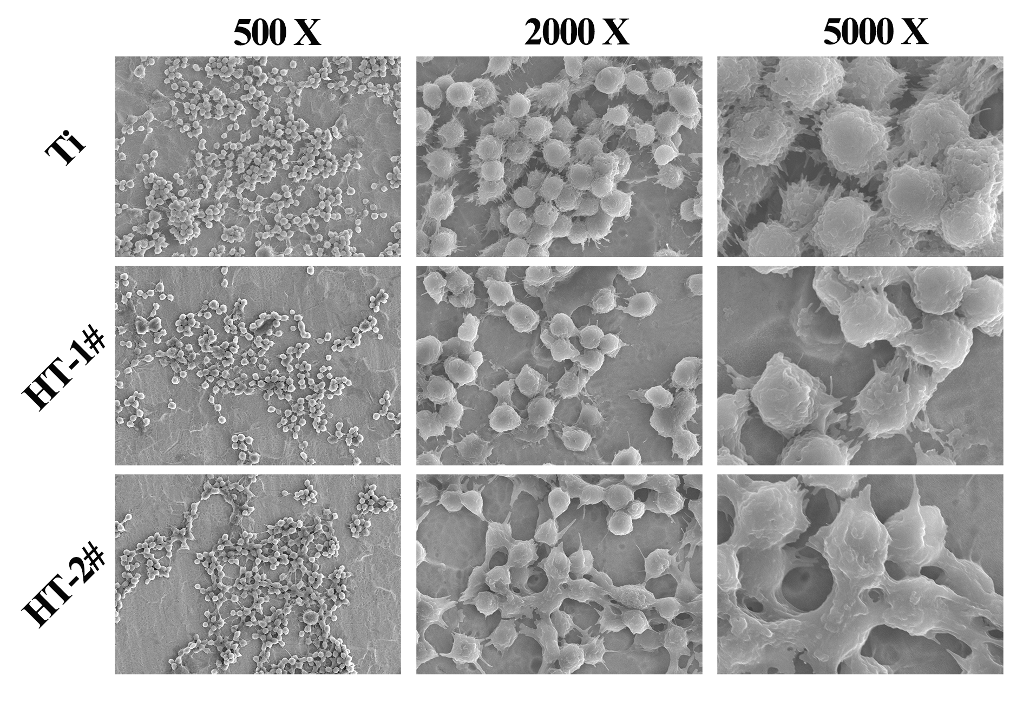


**Figure S2.** SEM images of BMDMs after cultured on various samples for 12 h.

**
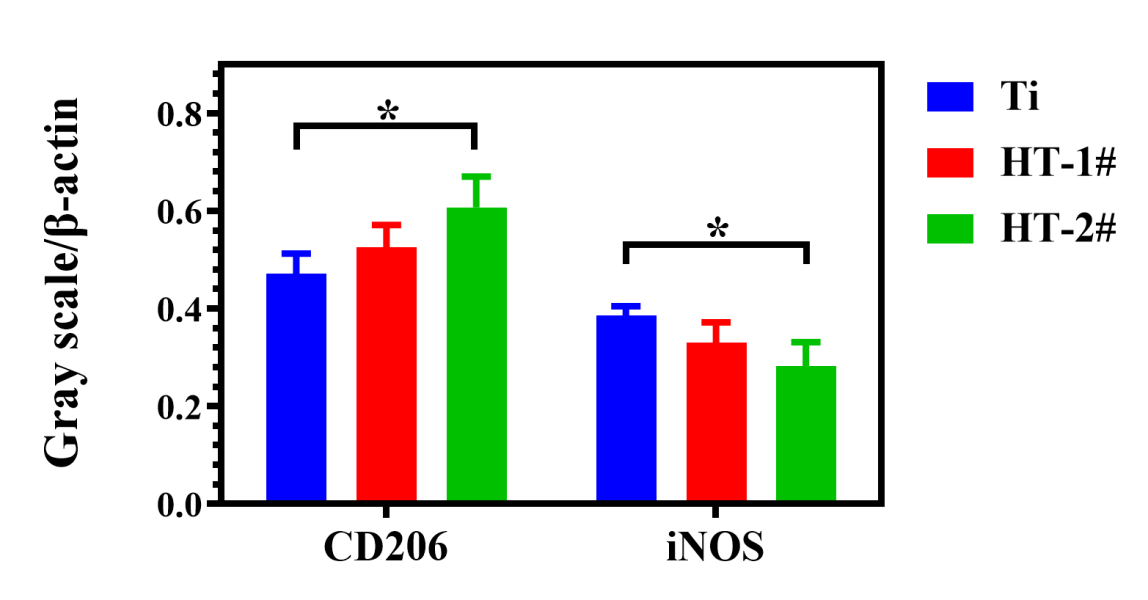
**

**Figure S3.** Quantitative analysis of the protein expression in WB assay.

**
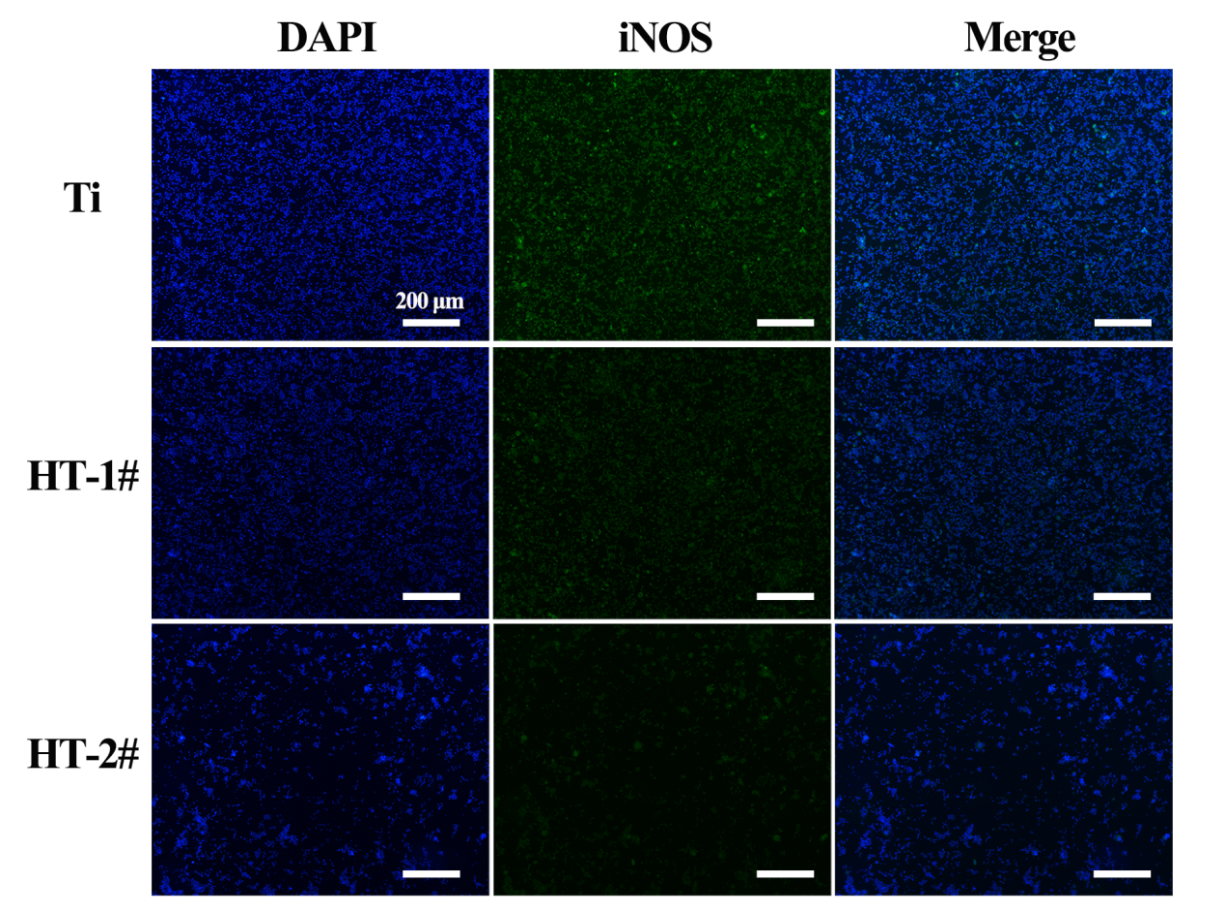
**

**Figure S4.** Immunofluorescence images of iNOS in BMDMs after cultured on various samples for 3 days.

**
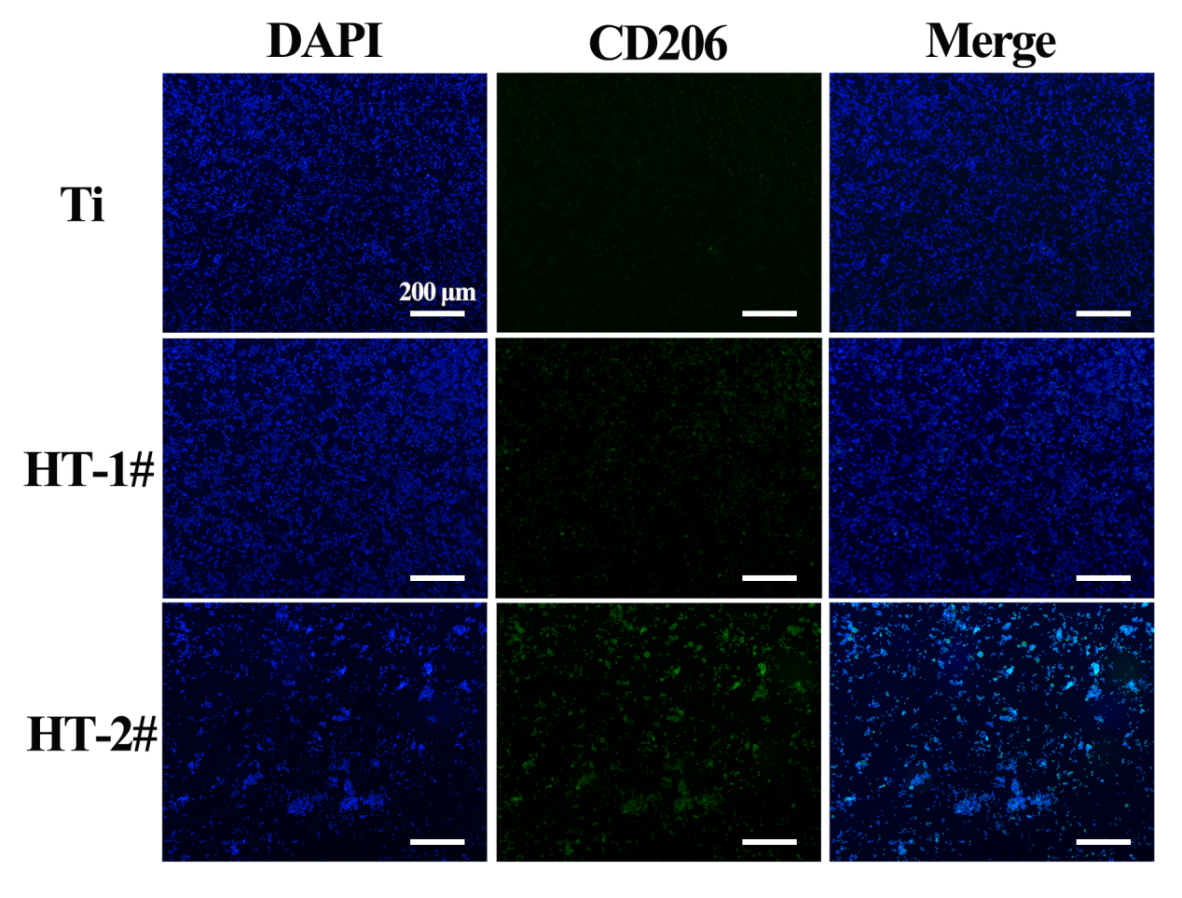
**

**Figure S5.** Immunofluorescence images of CD206 in BMDMs after cultured on various samples for 3 days.

**
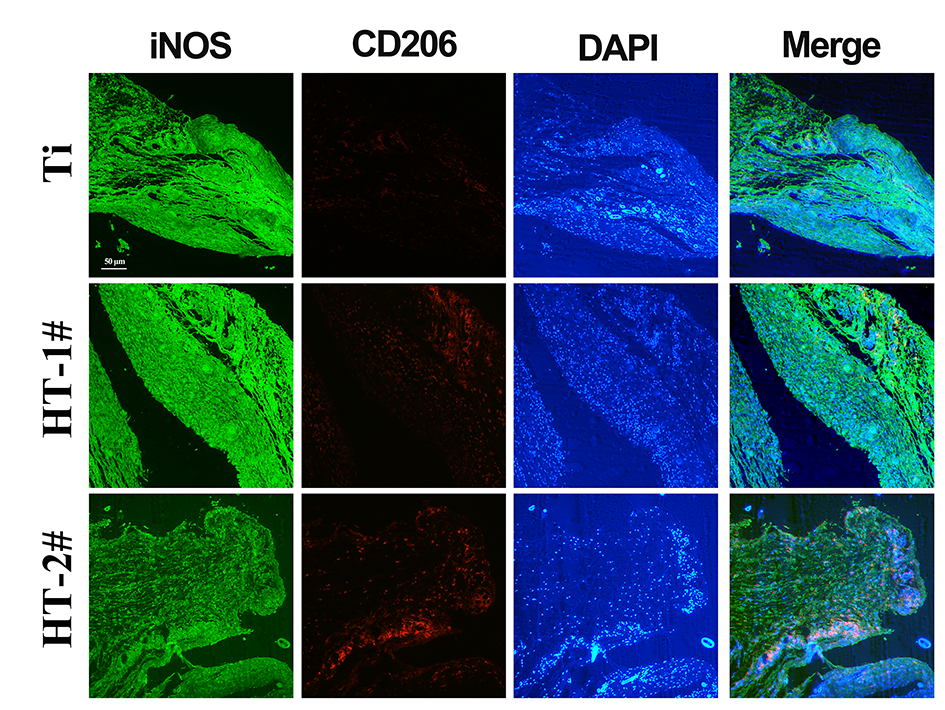
**

**Figure S6.** Enlarged immunofluorescence images of iNOS and CD206 in tissues adjacent to various samples.
